# Distinct roles for nitric oxide in resistant C57BL/6 and susceptible BALB/c mice to control *Burkholderia pseudomallei *infection

**DOI:** 10.1186/1471-2172-12-20

**Published:** 2011-03-16

**Authors:** Katrin Breitbach, Patimaporn Wongprompitak, Ivo Steinmetz

**Affiliations:** 1 Friedrich Loeffler Institute of Medical Microbiology, Ernst Moritz Arndt University Greifswald, Martin-Luther-Strasse 6, 17489 Greifswald, Germany; 2 Department of Immunology, Faculty of Medicine Siriraj Hospital, Mahidol University, Bangkok, Thailand

## Abstract

**Background:**

*Burkholderia pseudomallei *is the causative agent of melioidosis, an emerging bacterial infectious disease in tropical and subtropical areas. We recently showed that NADPH oxidase but not nitric oxide (NO) contributes to resistance in innately resistant C57BL/6 mice in a *B. pseudomallei *respiratory infection model. However, the function of NO for resistance was shown to differ among distinct strains of mice and proved also to be stage dependent in various infection models. The present study therefore aimed to examine the role of NO in a systemic infection model of melioidosis and to test whether the function of NO differs among innately resistant C57BL/6 and susceptible BALB/c mice after *B. pseudomallei *infection.

**Results:**

C57BL/6 *iNOS-/- *mice that were intravenously infected with *B. pseudomallei *survived several weeks, whereas most of the wild type animals succumbed during this period. The bacterial burden in liver and spleen was significantly higher in wild type animals compared to *iNOS-/- *mice 13 days after challenge. In contrast, BALB/c mice that were treated with amminoguanidine to inhibit NO expression in vivo showed significantly enhanced mortality rates and higher bacterial loads in liver and spleen compared to control animals. The bactericidal function of IFN-γ stimulated C57BL/6 *iNOS-/- *macrophages were not altered after *B. pseudomallei *infection, but BALB/c macrophages exhibited reduced killing activity against the pathogen when NO was inhibited.

**Conclusion:**

Our present data indicate a dual role of NO among resistant and susceptible mouse strains after *B. pseudomallei *infection. NO mediated mechanisms are an essential component to control the infection in susceptible BALB/c mice. In contrast, NO production in *B. pseudomallei *infected C57BL/6 mice rather harmed the host likely due to its detrimental effects.

## Background

Nitric oxide (NO) is a free radical molecule that can be expressed by several cell types including fibroblasts, hepatocytes, and phagocytes via nitric oxide synthases. NO exhibits many pleiotropic functions, among these microbicidal activity, and a role in immune regulation are of special interest after infection with parasites, bacteria or viruses. Release of NO can restrict the growth of several pathogens in the host [1-5], but is also known to cause nonspecific damage in host cells that can lead to an exacerbation after infection [2,6]. A rather protective or damaging function for NO was also described to be dependent on the stage of infection or the background mouse strain in several murine infection models [4,6-9].

*Burkholderia pseudomallei *comprises a facultative intracellular gram-negative rod and is the causative agent of melioidosis, an emerging infectious disease of humans and animals in certain areas of the tropics and subtropics [10]. The infection is thought to be acquired by inoculation into minor cuts, abrasions, or inhalation after contact with contaminated water or soil [11]. Clinical manifestations are variable, ranging from inapparent to localized chronic infections and fulminant acute septicemias with high mortality rate [12].

Several reports could show that the inbred mouse strains C57BL/6 and BALB/c differed in their outcome after *B. pseudomallei *challenge [13-15]. Irrespective whether mice were infected intravenously or via inhalation, the LD50 of C57BL/6 mice was 100-fold higher compared to BALB/c mice [13,14]. In addition, BALB/c mice exhibited significantly higher bacterial loads in liver and spleen already 12 h after intravenous infection compared to C57BL/6 mice [13]. Thus, C57BL/6 mice are considered to represent a relatively resistant mouse strain in murine melioidosis, whereas BALB/c mice are highly susceptible. We previously have shown that macrophages were essential for resistance in both BALB/c and C57BL/6 mice to control *B. pseudomallei *infection [16]. By further investigating bactericidal effector molecules of macrophages, we could not find any protective function for NO in resistance of C57BL/6 mice in a respiratory infection model or in controlling intracellular *B. pseudomallei *in macrophages [16]. However, we could neither exclude whether NO might play a role when bacteria were administered systemically, or a protective function in innate susceptible BALB/c mice.

The present study aimed to further elucidate the role of NO for resistance against *B. pseudomallei *in a systemic model of murine melioidosis. We compared the outcome of susceptible BALB/c and relatively resistant C57BL/6 mice lacking NO expression after intravenous infection with *B. pseudomallei*. To examine the impact of NO in controlling intracellular *B. pseudomallei *growth in macrophages, we analyzed the intracellular survival kinetics in primary bone marrow derived macrophages from BALB/c and C57BL/6 mice that lack NO release.

## Results

### Lack of iNOS renders C57BL/6 mice more resistant against systemic *B. pseudomallei *infection

**Figure 1:**
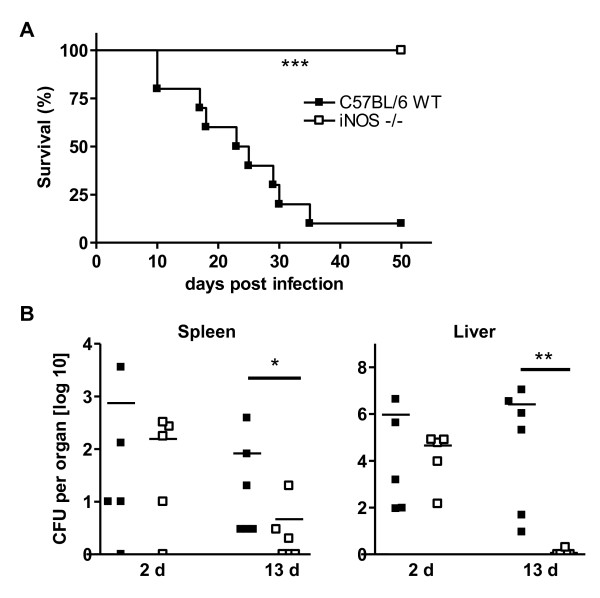
**Detrimental role of NO in innate resistant C57BL/6 mice after *B. pseudomallei *challenge**. (A) Survival curve of C57BL/6 wild type and *iNOS-/- *mice (n = 10) after intravenous (i.v.) infection with 0.2 - 1 × 10^5 ^CFU *B. pseudomallei*. Pooled data from two experiments are shown. (B) Bacterial burden in liver and spleen of C57BL/6 wild type and *iNOS-/- *mice 2 d and 13 d after i.v. infection with ~ 3 × 10^4 ^CFU *B. pseudomallei*. Pooled data from two independent experiments are shown. Each dot represents the bacterial count of the respective organ of a single animal. The line represents the median of each group. Statistical analyses were performed by using the Mann-Whitney test.

Nitric oxide (NO) and reactive oxygen intermediates are downstream effector molecules of IFN-γ that vary in their importance for resistance among a wide array of pathogens in murine infection models [3-5]. Whereas NADPH oxidase was crucial for resistance in C57BL/6 mice, we did not find any role for NO to control *B. pseudomallei *infection in the resistant mouse strain in a respiratory infection model. Moreover, C57BL/6 wild type mice even showed an increased bacterial burden compared to *iNOS*^*-/- *^mice [16]. To test whether NO might have a protective role after systemic infection with *B. pseudomallei*, we investigated *iNOS*^*-/- *^mice in an intravenous infection model of melioidosis. As demonstrated in Figure 1A, C57BL/6 wild type animals that received a moderate *B. pseudomallei *infection dose, died within approximately one month after challenge whereas all *iNOS-/- *animals survived the observed period. Significant differences in the bacterial load of liver and spleen were observed about 13 days after challenge (Figure 1B). These data indicate that, in accordance with our results obtained in the acute respiratory infection model, NO mediated functions seem to have rather detrimental than beneficial effects in resistant C57BL/6 mice after intravenous *B. pseudomallei *challenge.

### NO contributes to resistance against *B. pseudomallei *infection in BALB/c mice

**Figure 2:**
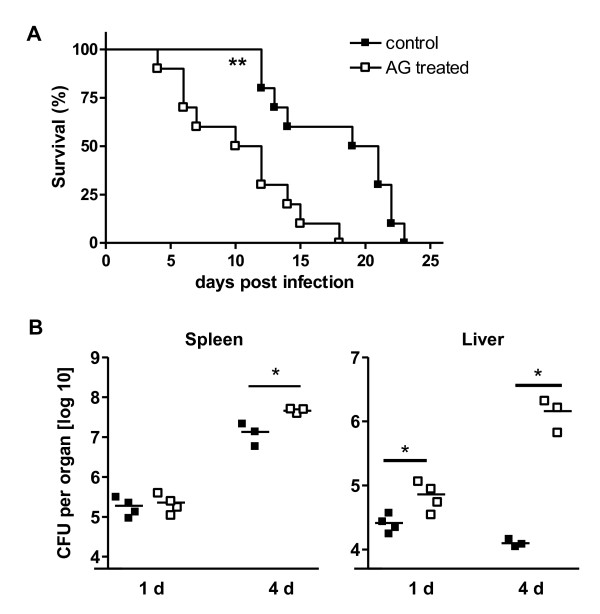
**Protective role of NO for resistance in innate susceptible BALB/c mice after *B. pseudomallei *challenge**. (A) Survival curve of AG-treated and control BALB/c mice (n = 10) after i.v. infection with ~300 CFU *B. pseudomallei*. Pooled data from two independent experiments are shown. (B) Bacterial burden in liver and spleen of sham treated and AG treated BALB/c mice 1 d and 4 d after i.v. infection with ~ 400 CFU *B. pseudomallei*, respectively. Data from single experiments are shown. Each dot represents the bacterial count of the respective organ of a single animal. The line represents the median of each group. Statistical analyses were performed by using the Mann-Whitney test.

In a murine *Toxoplasma gondii *infection model the contribution of NO for resistance was shown to differ among different strains of mice [7,9]. Since genetically iNOS deficient mice on BALB/c background are at present not available, aminoguanidine (AG) treatment was used to selectively inhibit NO release in BALB/c mice in vivo, an approach that has been successfully established in several murine infection models [4,7]. To show the effectiveness of AG treatment in vivo, serum of BALB/c mice that were intravenously infected with ~ 5 × 10^4 ^CFU *B. pseudomallei *was taken 24 h after infection. The concentration of nitrite in the serum of sham treated mice was 74.3 ± 7.2, and 38.4 ± 3.2 in AG treated animals (n = 5, *p *= 0.0018). Thus, release of NO in vivo was significantly reduced under AG treatment and *B. pseudomallei *infection. To examine the role of NO for resistance in susceptible BALB/c mice after *B. pseudomallei *infection, sham-treated and AG treated mice were intravenously infected with *B. pseudomallei*. Our data demonstrate that inhibition of NO significantly impaired survival rate of BALB/c mice compared to control animals (Figure 2A). By comparing the bacterial load of internal organs we found significantly enhanced bacterial burden within 24 h after infection in the liver of AG treated animals compared to control mice (Figure 2B). Four days after infection bacterial load was higher in both spleen and liver in NO depleted mice (Figure 2B). These data suggest that NO contributes to resistance against *B. pseudomallei *challenge in the susceptible mouse strain BALB/c.

### Macrophages from C57BL/6 and BALB/c mice lacking NO differ in their ability to control intracellular growth of *B. pseudomallei*

**Figure 3:**
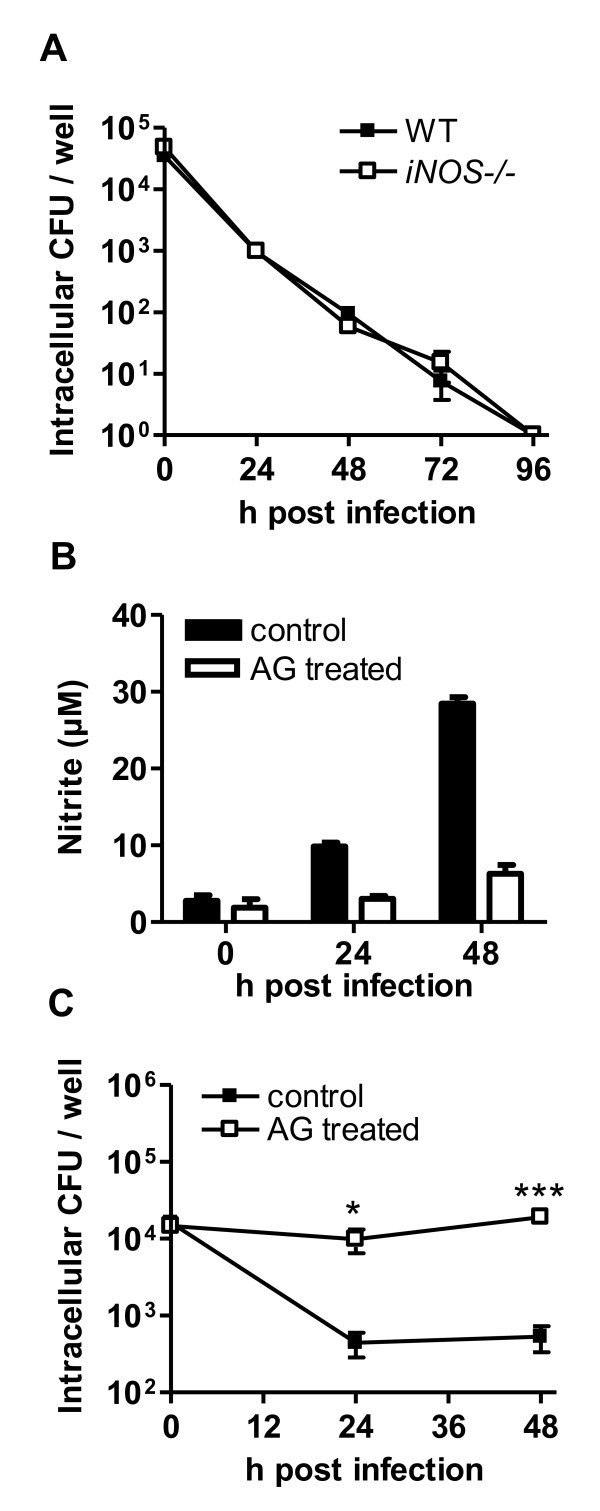
**Differential function for NO in macrophages from C57BL/6 and BALB/c mice to control *B. pseudomallei *growth**. (A) Intracellular survival of *B. pseudomallei *in IFN-γ stimulated BMM from C57BL/6 wild type and *iNOS*-/- mice (MOI ~ 50:1). One representative experiment out of five independent experiments is shown. (B) NO production of BALB/c macrophages without and with AG treatment after *B. pseudomallei *infection (MOI ~ 65). (C) Intracellular survival of *B. pseudomallei *in IFN-γ stimulated BALB/c BMM (MOI ~ 65) with and without AG treatment. One representative experiment out of three independent experiments is shown. Values are the means ± standard deviations from triplicate determinations. Statistical analysis was performed using the Student's t-test.

In our previous study we showed that primary bone marrow derived macrophages obtained from resistant C57BL/6 mice exhibited a better ability to eliminate intracellular *B. pseudomallei *compared to macrophages from susceptible BALB/c mice [16]. This phenomenon became especially apparent after IFN-γ stimulation, suggesting that IFN-γ mediated effector functions are crucial for determining the distinct bactericidal activity of these cells. Since we found that NO differs in its role to control *B. pseudomallei *infection among resistant and susceptible mice in vivo, we were interested whether NO might also differ in its contribution to eliminate intracellular *B. pseudomallei *in macrophages from C57BL/6 and BALB/c mice. We therefore examined the intracellular survival kinetics of intracellular *B. pseudomallei *in iNOS deficient C57BL/6 macrophages. We could not detect any role for NO to control intracellular *B. pseudomallei *growth in macrophages from relatively resistant C57BL/6 mice, even by extending the incubation time up to four days (Figure 3A). To test the role of NO in macrophages from more susceptible BALB/c mice, macrophages were treated with AG in vitro to inhibit NO release. As shown in Figure 3B, AG treatment could reliably inhibit NO release from *B. pseudomallei *infected macrophages. In contrast to the results obtained in NO deficient C57BL/6 macrophages, BALB/c macrophages in that NO was inhibited could not reduce the number of intracellular *B. pseudomallei *within 24 h and 48 h after infection compared to untreated macrophages (Figure 3C). These results are consistent with our data obtained in vivo, in that NO has beneficial and bactericidal effects in BALB/c mice, underlining the importance for NO in the susceptible mouse strain.

## Discussion

The IFN-γ effector molecule NO is known to contribute to resistance against a variety of pathogens such as *Mycobacterium tuberculosis*, *Salmonella, Chlamydia, Toxoplasma gondii*, and others [1-5]. Our present data indicate that NO seems to have a two-sided role in murine *B. pseudomallei *infection, dependent on the background mouse strain. In a respiratory infection model of melioidosis we recently reported that NO did not play a role for resistance in innate relatively resistant C57BL/6 mice, but *iNOS-/- *mice tend to be more protected from infection [16]. Similar observations were obtained in mice that were challenged intravenously with *B. pseudomallei *in the present study. Similar phenomena have also been observed in a murine *Toxoplasma gondii *and HSV-1 infection model [6,17] where inhibition of NO led to prolonged survival of infected mice. Since at the same time lack of NO expression was associated with an increased load of the respective pathogen in tissues, it was suggested that NO has both, a microbicidal function as well as a detrimental role that led to immunopathologic changes and finally caused death [6,17]. However, the bacterial load in internal organs of C57BL/6 *iNOS-/- *mice was neither enhanced in a respiratory nor in an intravenous *B. pseudomallei *infection model. In contrast, the number of live bacteria was significantly lower compared to control animals ([16] and present study). This is in clear contrast to results obtained in *Toxoplasma *and HSV-1 infection studies where NO - despite its damaging effect - had a role in restricting growth of the pathogens [6,17]. However, after infection with *B. pseudomallei*, dispensable NO production in wild type mice is likely to cause damage to host cells. This might eventually led to a reduced ability to control bacterial growth and finally contribute for a higher mortality compared to animals that lack NO expression. Thus, in contrast to other murine infection models in that NO beside its detrimental effects still exhibit detectable killing activity against pathogens [6,7], we did not found NO to contribute for the elimination or even reduction of the bacterial load after *B. pseudomallei *infection in innate relatively resistant C57BL/6 mice.

In contrast to the phenotype observed in C57BL/6 mice, we found NO to contribute to in vivo resistance in innate susceptible BALB/c mice. Also the in vitro killing activities of primary BALB/c macrophages were significantly decreased when NO was inhibited. Since our previous finding revealed that macrophages are absolutely essential for resistance in both susceptible and resistant mice [16], we assume that NO-mediated killing activity of macrophages is likely to contribute to resistance in BALB/c mice. This assumption is supported by our finding that enhanced mortality was associated with enhanced bacterial burden in spleens and livers of AG treated BALB/c mice. The fact that NO was dispensable for controlling *B. pseudomallei *growth in C57BL/6 mice indicates that IFN-γ dependent but NO-independent bactericidal mechanisms are crucial for resistance in this mouse strain. In contrast, bactericidal mechanisms that are present in C57BL/6 mice obviously lack to work efficiently in BALB/c mice. Thus, beneficial effects of NO seem to outweigh damaging effects in susceptible mice after *B. pseudomallei *challenge. Differences in the role of NO for resistance in susceptible and resistant mice have so far been described in a *Toxoplasma gondii *infection model, where - similar to our findings - NO contributed to resistance in innate susceptible but not resistant mice [7,9].

## Conclusions

The function of NO in resistance against a pathogen can differ among susceptible and resistant mouse strains. We here report that NO has rather damaging effects in innate resistant C57BL/6 mice in a murine model of melioidosis and did not play any role in the killing activity of C57BL/6 macrophages. In contrast, NO contributed to full resistance in innate susceptible BALB/c mice and was also involved in restricting the intracellular growth of macrophages from susceptible mice. The ambiguous role for NO to either protect or affect the host is likely to serve as one component that determines susceptibility against *B. pseudomallei *infection.

## Methods

### Bacteria

*Burkholderia pseudomallei *strain E8 was used throughout the study [16]. Bacteria were grown on Columbia agar at 37°C for 24 h. Colonies were harvested using LB broth containing 20% glycerol and frozen in 50 μl aliquots at -70°C.

### Animals

Female BALB/c mice were obtained from Charles River (Wiga Sulzfeld, Germany). *iNOS-/- *and respective C57BL/6J control mice were obtained from The Jackson Laboratory (Bar Harbor, Maine). Animals were provided with food and water ad libitum. Generally, naturally occurring death of infected animals was documented as survival end points. However, approx. 20 percent of mice that were observed to be severely ill were euthanized. All mouse experiments were performed in full compliance with the relevant laws and approved by the Landesamt für Landwirtschaft, Lebensmittelsicherheit und Fischerei, Western Pommerania, Germany (Az: LALLF M-V/TSD/7221.3-1.1-044/05).

### Infection of mice and determination of organ bacterial burden

Frozen bacteria were thawed from the stock, plated on Colombia Agar, and grown overnight. Prior to infection, bacteria were harvested from Colombia Agar and diluted in phosphate buffered saline (PBS) to the appropriate concentration. For intravenous (i.v.) infection, a bacterial suspension of 200 μl was injected into the lateral tail vein. The mortality of the animals was monitored daily. To enumerate bacteria in internal organs, organs were aseptically removed and homogenized in 0.5 to 1 ml sterile PBS containing 0.5% Tergitol and 1% bovine serum albumin. Organ suspensions were plated on Ashdown agar and number of CFU was determined.

### NO inhibition in vivo

To selectively inhibit NO production in vivo, we treated mice with aminoguanidin (AG) as reported by others [18]. Mice continuously received AG via drinking water (2% w/v) at least six days prior to and during infection and were additionally treated i.p. 6 h prior and 18 h after *B. pseudomallei *challenge with AG (1.5 mg AG per animal in 200 μl PBS). Control animals received sham injections with PBS.

### Culture and infection of bone marrow derived macrophages (BMM)

Murine bone marrow derived macrophages (BMM) were generated under serum-free conditions from BALB/c mice as previously described [19]. Briefly, tibias and femurs were aseptically removed, and bone marrow cells were flushed with sterile PBS and then centrifuged at 150 × *g *for 10 min. Cells were resuspended in RPMI medium containing 5% Panexin BMM (PAN Biotech, Aidenbach, Germany), 2 ng/ml of recombinant murine granulocyte-macrophage colony-stimulating factor (PAN Biotech), and 50 μM mercaptoethanol and cultivated for at least 10 days at 37°C and 5% CO_2_. Twenty-four hours prior to infection experiments, cells were seeded in 48 well plates (1.5 × 10^5 ^cells per well) and infected with *B. pseudomallei *at a MOI as indicated for each experiment for 30 min. Cells were then washed twice with PBS and medium containing 100 μg/ml kanamycin was added to each well. At the indicated time points (time zero was taken 20 min after incubation under antibiotic-containing medium) the number of intracellular colony forming units (CFU) was determined[19]. IFN-γ stimulation was performed using 100 U/ml IFN-γ. For in vitro NO inhibition of BALB/c BMM, cells were treated by adding 2 mM AG into the medium 16-20 h prior to infection, during the 30 min infection time with *B. pseudomallei *and subsequent incubation under kanamycin-containing medium.

### NO-Assay

Measurement of NO was performed as recently described [19]. To measure NO in cell culture supernantant, cells were seeded in 96 well plates 24 h prior to infection and IFN-γ at a concentration of 100 ng/ml was added. Cells were infected with *B. pseudomallei *und incubated under kanamycin-containing medium as described above. At indicated time point cell culture supernatant was collected and NO production was determined by the Griess reagent system. To determine NO concentration in the serum, 100 μl serum samples were rigorously mixed with 100 μl acetonitril and centrifuged for 10 minutes at 10 000 × g to remove serum proteins. Serum supernatant was taken and measured as described above.

### Statistical analysis

Kaplan-Meier survival curves and the log rank test were used to compare the mortality of different groups of mice. To compare statistical significances of bacterial loads in internal organs or macrophages, either the Student's t-test or Mann-Whitney test was used as indicated for each experiment. Statistical significances were determined as follows: *p = 0.1, ** p = 0.01, *** p = 0.001.

## Authors' contributions

KB and PW performed and analyzed the experiments. KB and IS designed the study and wrote the manuscript. All authors have read and approved the final version of the manuscript.
